# Two Cases of Feigned Homicidality: Assessing the Third Dimension in Homicidal Threats

**DOI:** 10.3390/healthcare10010031

**Published:** 2021-12-24

**Authors:** Pavan Madan, Alexander Graypel, Alan R. Felthous

**Affiliations:** 1 Mindpath Health, Davis, CA 95616, USA; 2 Community and Long-Term Care Psychiatry LLC, Saint Louis, MO 63128, USA; agraypel84@gmail.com; 3 Department of Neurology and Psychiatry, Saint Louis University, Saint Louis, MO 63103, USA; felthous@slu.edu

**Keywords:** homicidal, case series, malingering, feigned, threat, risk assessment, psychiatric evaluation

## Abstract

Although data and research on the topic are lacking, the phenomenon of feigned homicidality in short-term hospitalization appears to have increased in recent years. Inpatient psychiatrists not only assess the seriousness of homicidal threats, but also whether such threats are authentic. However, specific literature and diagnostic manuals provide virtually no clinical guidance for this. The authors present two case examples of homicidality feigned for self-serving purposes that had little to do with hostility against the would-be victim. They recommend an approach to assessment that first takes any threat of homicide seriously, and involves an attempt to assess the seriousness of the threat and risk of harm. Secondly, if feigned homicidality is suspected, clinicians can methodically assess for this using criterion that have been applied to the assessment of malingering.

## 1. Introduction

The professional literature on assessment of a patient’s verbal threat to kill concerns a determination as to whether the homicidal statement is only a fantasy, not likely to be carried out, or a serious intention with a realistic potential for a lethal or injurious outcome. Not usually included in such discussions is a third possibility: the verbal statement is made to achieve a self-serving but non-homicidal goal. In many cases, the possibility of feigned homicidality must be considered together with fantasized and planned homicide when assessing risk. Without research and scientific literature on feigned homicidality, the clinician is faced with a daunting task when a patient threatens to kill another person.

Sixty years ago, it would have been rare for anyone to threaten to kill another person simply to gain hospital access. Times were different. Involuntary hospitalization in a mental facility could extend for months or years. Homelessness of the mentally ill was limited, and addiction to street drugs was not as prevalent. In recent years, increasing reference is made to the patient who achieves hospitalization for “three hots and a cot”, rather than for psychiatric treatment of symptoms of mental illness [1]. Without mentioning feigned homicidality specifically, literature on malingering recognizes hospitalization as a goal in some cases. However, feigned homicidality seems to be becoming a more frequent strategy for gaining hospitalization for various reasons.

Patients often have alternative motives for entering hospitals, such as the motive to detoxify comfortably from addicting drugs, to seek sedative medications, to obtain lodging that is more comfortable than a homeless shelter, or to avoid jail detainment. Intended escape from destructive conditions and circumstances is a positive motivation that is adaptive and supportive of mental health. Once detoxified, the addicted patient no longer claims suicidal or homicidal thoughts and may even admit to having fabricated such thoughts in order to gain hospitalization. Within a few days, they are eager to return to the situation from which they came without considering rehabilitative measures.

In this article, we present two cases of feigned homicidal ideations to gain access to hospital. We then discuss the recommended evaluation of verbal threat, assessment of risk, differentiation from pseudologia fantastica, and approach towards cases with suspected feigning of homicidal ideation. We hope that the cases and the discussion provide the reader with an understanding of how such cases may present at different stages of mental health treatment in a hospital, and when feigned homicidality is suspected, how the providers can perform a reasonable assessment to establish a diagnosis and treatment plan. We use two unrelated cases as examples. Both cases are discussed at length below, and their main findings are later summarized in Table 1.

## 2. Case Examples

### 2.1. CASE I

Mr. A was a Caucasian man in his forties with a history of chronic medical conditions and multiple emergency room (ER) visits for chest pain and alcohol intoxication. He was homeless and unemployed, having recently lost his job as a cook. He had no documented past psychiatric history. He was brought to the ER by emergency medical services after complaining of chest pain and possible overdose. Mr. A reported to the ER physician that he was having suicidal ideation and planned to drink excess amounts of alcohol, hang himself, or shoot himself with a gun, which he said he did not possess. The patient presented symptoms of depressed mood, hopelessness, helplessness, and decreased interest in things as well as decreased sleep. He reported a pending DUI charge, regular alcohol use with blackouts, buildup of tolerance, and withdrawal tremors. He denied symptoms consistent with mania. He denied hearing voices or having visions and showed no signs of response to internal stimuli.

The patient had hypertension, bilateral upper extremity neuropathy, COPD, and angina. He also reported a history of a stroke 6 years earlier, which led to loss of function in his right arm and left leg. Per neurology consultation, this stroke was very unlikely, and the patient had no current stroke-related deficits.

On the mental status examination, he was hostile and irritable during the interview. He described his mood as “very sad at this point”. Thought content included suicidal ideation with a method. He denied experiencing voices or visions. He was alert and oriented to place and time and appeared to have medical decision-making capacity, but he refused to cooperate with the mini-mental status exam.

On the first day after admission, the patient said he slept poorly; however, the nurses reported that he had slept for 8 hours. He showed no withdrawal symptoms. He continued to express suicidal ideation without any plan. He denied having homicidal thoughts towards anyone. On the second day, he was angry over the food, the nurses, other patients, the medical students, and at the unit in general. He did not attend any of the group therapy sessions, was aggressive during interviews, and reported his mood to be “terrible”. He also started complaining of voices telling him to hurt himself; however, he was never seen responding to internal stimuli.

Over the next few days, the patient continued to report suicidal ideation with various methods, ranging from hanging to not having a method. He became more social and attended some groups. During one interview, he revealed that he had homicidal ideation towards five individuals outside the hospital, but he declined to provide any information about them. He displayed narcissistic traits, especially when criticizing the hospital food, often saying “I can cook around these fools”. He described his desire to work in a fancy restaurant one day, which was inconsistent with his talk of abruptly bringing about his own death.

When asked for consent to talk to his family, he said that he would allow his doctors to talk to his brother, but only after he talked with him first. Immediately after sharing that his brother was an important government employee, he described elaborate revenge fantasies towards three individuals, who he said had wronged him in the past. One of the individuals was a previous employer, the other individual was his ex- girlfriend’s mother, who he said he wanted to torture and strangle. This was to retaliate for causing him and his girlfriend to break up. The third individual used to be his best friend, but then this man impregnated his ex-girlfriend. He claimed that he thought this because he knew his friend’s blood type and that of his own, and believed that the baby’s blood group corresponded with that of his best friend and his ex-girlfriend’s, and therefore, that the baby must have been fathered by the other man. There were two more people who the patient said he had thoughts of killing, but he refused to tell his treatment team about them. He exclaimed dramatically, “I will tell you when the time is right!” Over the next few days, Mr. A attended all of the groups, ate all of his meals, and was cooperative with medication.

Mr. A spoke more about his “revenge plans”, which were very unrealistic and sounded like a revenge fantasy movie. He said that he did not expect to get caught because he was “just too good”. One of the authors spoke with Mr. A’s brother, who confirmed the patient’s history of alcoholism. His brother was unsure about any “revenge plans” and could not confirm any of the events that the patient described. However, he shared that his brother had been emotionally hurt by his ex-girlfriend in the past, but he had not heard about any paternity situation. His brother said that the patient had a good support system on the outside and many family members; however, many of them have children, and they did not want Mr. A around them when he was drunk. Once Mr. A becomes abstinent, he would have their full support.

Mr. A then developed plans to enter an alcohol rehabilitation program close to home so he could spend more time with his family. When asked again about homicidal thoughts, he described them but was inconsistent and less detailed than before. He again talked about his ex-girlfriend, but this time, he appeared to have forgotten what he had reported to his treatment team a few days earlier and said that he was very depressed because his ex-girlfriend had died in a car accident with the baby still inside of her. There were several inconsistencies in his story, and his brother also confirmed that the ex-girlfriend was still alive despite the patient having said that she died in a car accident. Other homicidal thoughts continued to be vague, and he continued to alter the number of people he wanted to kill. Citalopram and trazodone were discontinued and replaced with mirtazapine to help with his chronically poor sleep, appetite, and mood. The patient placed a telephone call and planned to go into rehabilitation.

A few days later, the patient asked the physician if he could have a doctor’s note confirming his stay in the hospital with the exact dates of hospitalization. He said he needed the note because he had missed two court cases that were scheduled two days following admission, and that he was unable to attend them as a result of being in the hospital. He added that this had nothing to do with the reason that he presented to the hospital.

The next day, the patient said his homicidal thoughts were only towards two people. He was still irritable on the unit and complained about the food and other patients. However, he was social with his peers, laughing, playing cards, and joking at times.

As the time neared the patient’s rehabilitation intake date, he began to deny suicidal ideations. He continued to express homicidal ideation, but these thoughts were assessed as likely revenge fantasies or malingering given their inconsistencies and vagueness.

The patient was contacted by one of the authors 3 months after his discharge to see how he was doing. He said that he was doing great and maintaining his sobriety. When asked about his homicidal thoughts, the patient said, “That’s the furthest thing from my mind”.

The diagnosis of feigned homicidality was based on (1) inconsistent history over time; (2) inconsistency between Mr. A’s account from that of his brother; (3) evidence of a clear external incentive for hospitalization; (4) no clear plan for homicide or access to a weapon, and; (5) lack of cooperation with the diagnostic assessment.

### 2.2. CASE 2

Mr. B was a 46-year-old African American divorced male, with a history of schizoaffective disorder and hypertension, who presented to the ER with complaints of “hearing voices and feeling suicidal and homicidal”. The patient told the ER physicians that he was hearing voices telling him to jump off a bridge. He said that, one week before, on being commanded by voices, he had jumped off the balcony from his second floor and suffered minor knee abrasions. When the psychiatry resident interviewed Mr. B, he said that the voices were telling him to jump off the roof of his two-story apartment (but he did not mention the bridge). He said that he was hearing male voices telling him to hurt his sister and kill her. He said that he was upset with his sister due to a will dispute, which arose after the death of their mother 2 years previously. He alleged that his sister took money from the inheritance to open a business, and that this incident had infuriated all of his siblings. Mr. B stated that he did not see her on a regular basis, but knew where she lived and how to reach her.

Mr. B reported several depressive symptoms including feeling depressed, poor sleep, poor appetite, 15 lbs. weight loss, feelings of loneliness, hopelessness, anger at God for taking away his mother, poor concentration, and lack of interest in pleasurable activities. He also reported feeling nervous. He reported feeling paranoid that “people are out there to get me”.

Mr. B reported that he had been previously diagnosed with schizophrenia, bipolar disorder and schizoaffective disorder. He stated that he first started hearing voices at the age of 32 years, after the death of his father. He had been taking antipsychotics off and on for the past several years. Previous psychiatric hospitalization revealed the diagnoses of cannabis abuse, cocaine abuse, and substance-induced mood and psychotic disorders. The patient reported having had multiple psychiatric hospitalizations in the past, including one in the same hospital as the current presentation. He reported to have made one suicide attempt 4 years earlier by overdosing on his medications “because the voices told me to do so” (he did not report any suicide attempt 1 week previously).

The patient reported being treated with oral as well as long-acting risperidone injection during his previous hospital stay, along with sertraline and trazodone. These were confirmed in the medical records obtained from another facility.

Mr. B admitted to smoking a pack of cigarettes daily, drinking 12 beers a day, smoking a bag of marijuana daily, and using cocaine occasionally. Mr. B said that he lived by himself in an apartment. He did not work and received financial assistance from the state for his disability, i.e., schizophrenia and bipolar disorder.

From previous hospitalization records, the patient had been admitted to this hospital with complaints of hearing voices that told him to kill himself in the context of recent use of alcohol, cocaine, and marijuana 2 months before this presentation. The next day, when it was discovered that a female patient on the unit was related to him, the patient was told that he had to be transferred to another hospital. At this point, the patient became upset and said that he was not hearing voices anymore, nor was he feeling suicidal or homicidal and did not want to go to another hospital.

Mr. B said that he was no longer hearing voices, but he continued to feel suicidal as well as homicidal (against his sister). He denied having any means with which he could kill her, and this was the only factor that prevented him from killing her. When questioned regarding the possible consequences of killing his sister, Mr. B responded that he was confident that he would not go to jail because his entire family would support him in his act. When gently confronted with the idea that his family support may have little to do with the legal accountability of his behavior, he became irritable, and the conversation could not be pursued further. His affect remained flat throughout this interview.

On the second day of hospitalization, Mr. B was seen to be out and about on the unit, interacting with peers and staff members and attending all group therapy sessions, but continuing to express homicidal ideation.

On the third day, the patient’s homicide risk was assessed using the method suggested by Borum and Reddy [2]. (1) Attitudes: The patient did not have any history of prior violence. His family history did not support violence. He continued to pray. (2) Capacity: He denied having considered a method of homicide, so capacity could not be adequately assessed. He said, however, that he was going to move to Florida (from the Midwest) to remove himself from his sister, which would decrease opportunity/capacity. (3) Threats: Although Mr. B had complained to his sister and her coworker in the past about her stealing the money from their inheritance, he denied having threatened her, having been physically aggressive towards her, or having done anything to prepare to harm her. (4) Intent: He denied current intent. (5) Others: He said that his family may support him if he killed her, as they were also angry at her for stealing, but he did not feel that they would join him or encourage him in doing so. (6) Noncompliance: He appeared to be compliant with risk reduction measures. Additionally, he denied access to a weapon. He provided his physician verbal permission to contact his sister. He finally said that he did not want to kill her. From this assessment, the final impression was that the risk of violence was low and that his physicians did not have sufficient reason to either notify police or pursue involuntary hospitalization. After his sister was contacted and notified by the resident about his expressed feelings and violent thoughts, Mr. B was discharged from the hospital to enter a substance abuse inpatient rehabilitation facility.

After the patient’s discharge, some of his belongings were discovered in the possession of the female patient on the unit. When she was confronted, she confessed that she was Mr. B’s “significant other” (with a different last name) and they had arrived in the ER together but had not disclosed their relationship (as they had not been allowed to stay together on the same unit during their previous visit to this hospital). Moreover, they had decided to simultaneously feign similar psychiatric symptoms of hearing voices and feeling suicidal and homicidal to gain admission because they were homeless and needed a place to stay for a week. This confession provided a rare confirmation of the suspicion that the symptoms of homicidality were feigned for external incentive. In retrospect, the Borum factors proved to be helpful in accurately evaluating low risk of harm in this case.

Homicide risk assessment supported feigned homicidality based upon the following. (1) The patient was known to feign similar symptoms for a clear external incentive (housing) during the previous two visits; (2) he was currently experiencing an acute need for housing; (3) he had not been compliant with his medications for several months but had not “relapsed” until 3 days previously; (4) Mr. B denied knowing the female patient, with whom he had presented to the same ER during his past two visits; (5) the patient did not have easy access to a weapon; (6) the patient had planned to move to Florida after 2 weeks; and (7) he was not experiencing acute psychotic symptoms. The homicide risk was assessed to be low, and the patient was discharged from the ER after medical stabilization for hypertension.

## 3. Discussion

The cases highlight several important clinical points towards the assessment of feigned homicidality. Keeping these two cases in mind, we would now like to discuss how to differentiate feigned homicidality from pseudologia fantastica, how to evaluate a verbal threat, how to assess homicidal risk, and eventually how to conduct further assessment when feigned homicidality is suspected We then discuss the duty to protect and the use of a multidisciplinary approach in the management of such cases.

### 3.1. Differentiating Feigned Homicidality from Pseudologia Fantastica

Today, pseudologia fantastica is not to be found in the DSM 5 [3], although factitious disorder is, earlier known as Munchausen’s Syndrome. The original story of Van Munchausen was of a legendary baron who told fanciful false episodes of adventure. Kraepelin referred to Munchausen when describing psychological processes of “hunter traits”, which he considered within the realm of normalcy. Delbruck [4] was the first to use the term “pseudologia fantastica” in 1891 and describe it as a phenomenon that is observed in normal, characterologically disturbed, as well as mentally ill individuals. It exists on a continuum between lying with full awareness of the statement’s untruths and delusions where the unreality of the belief is not appreciated. In pseudologia fantastica, the subject’s vulnerability to self-deceit from his own falsehoods can vary and fluctuate.

In summarizing the features of pseudologia fantastica from the literature, Birch and colleagues [5] list “excessive, impulsive lying” that usually begins in adolescence and then persists. The lies are fanciful, easily shown to be falsehoods, and are self-destructive. Motives are internal such as wishful fantasy, and not externally motivated, such as to gain materially or avoid punishment. Pseudologia fantastica involves impaired reality adherence but not to the degree found in delusions. More recently, Thom et al. [6] described the phenomenon of pseudologia fantastica as a state of chronic lying/storytelling that is dramatic, seeking admiration or sympathy, and out of proportion to the obvious benefit.

### 3.2. Evaluating the Verbal Threat

Regardless of whether a homicidal threat is initially thought to be idle or serious, feigned or authentic, it should be assessed. Not many years after the California Supreme Court’s Tarasoff ruling in 1976 [7], Appelbaum [8] provided a three-step approach for dealing with homicidal threats that remains today as prudent guidance. Although the screening is derived from early Tarasoff-like case laws, this three-step approach seems self-evident in its practicality for any clinical risk assessment including the risk of suicide assessment: (1) Gather data that are relevant to the assessment of dangerousness. (2) Based upon this data, make a determination of dangerousness and select a course of action and (3) implement this plan. By case examples, Appelbaum illustrates how clinicians overreact and issue unnecessary warnings following insufficient assessment of the threat. Here, it is suggested additionally that assessment informs the action to be taken not only by a methodical determination of the patient’s level of seriousness and dynamics of the risk, but also by determining whether the threat is authentic or feigned. Whether a verbal threat of homicide is feigned is also relevant to its seriousness.

In theory, as with risk assessments in general, probability predictions that a homicidal threat will be carried out should be more comprehensive than just to answer forced, dichotomous questions such as whether to hospitalize or whether to issue protective warnings [9]. To address such protective actions to be taken, the assessment of the risk, whether associated with a verbal threat or not, should incorporate four inquiries [8]: (1) Is the patient dangerous to others? (2) Is the danger due to serious mental illness? (3) Is the danger imminent? and (4) Are potential victims of the danger reasonably identifiable? The present discussion focuses only on those patients who would be hospitalized either because the danger is due to serious mental illness in need of intensive treatment, or the danger is imminent and no less restrictive means of protection are available.

Whenever practical, it is helpful to explain to the patient at the beginning of the clinician–patient relationship the terms of confidentiality, including the possible exception of protective warnings. Assessment of the potential for externally directed violence should then proceed along with the assessment of the potential for self-harm and suicide. Family members, previous treatment providers, and other collateral sources should be contacted where doubt exists about the seriousness of an expressed threat [6]. As with the assessment for suicide, any initial threat of homicide is taken seriously and evaluated accordingly, even when the clinician suspects that the threat is feigned.

### 3.3. Assessment of Risk

With the realization that research on assessing the seriousness of homicidal threats is much less developed than research on the risk of aggressive behavior in general, the clinician can usefully apply the guidelines provided by Borum and Reddy [2] for assessing the seriousness of the risk of homicide. Practical, methodical, and literature-based, the Borum and Reddy factors are summarized with the acronym ACTION: (1) Does the patient hold Attitudes that support violence as a method to deal with the interpersonal conflict? (2) Does he have the Capacity to carry out the threat? There are several components to capacity: availability of the intended victim, availability of means (e.g., weapon), and physical capacity as well as mental capacity. (3) Has he already crossed Thresholds in the direction of violating the victim’s autonomy and privacy or of preparing to act violently against the victim (e.g., stalking, verbal threats, inappropriately accosting the victim at the victim’s place of employment)? (4) Does the patient have a serious Intent behind the threat? Is it conditional and will only be carried under specific conditions that are not yet in place? (5) Do the attitudes and reactions of significant Others support violent responses? Conversely, do family members and close friends favor non-violent approaches to interpersonal problems? (6) And finally, is the patient Non-compliant with attempts at risk reduction, such as following treatment recommendations, helping to identify and contact the victim (if a protective warning is indicated), and neutralization of firearms. The reverse, or absence of the Borum factors, can be considered as potentially protective against enactment of the threat. Ultimately, the determination of high, medium, and low risk for violence following a verbal threat of homicide is a matter of clinical judgment, and not numerically quantitative.

### 3.4. Assessment of Feigned Homicidality

If the suspicion of feigned homicidality persists even after the homicidal threat has been assessed, it should be further explored. Psychiatrists need more than just a gut feeling to determine if the patient is lying. Psychiatrists must be aware of their countertransference while conducting further assessment. Guidelines for malingering can be useful in this regard, with malingering being “the intentional production of false or grossly exaggerated physical or psychological symptoms, motivated by external incentives such as avoiding military duty, avoiding work, obtaining financial compensation, evading criminal prosecution or obtaining drugs” [3]. The clinician is challenged with a dearth of professional literature on the assessment of feigned homicidality and by the patient’s disinclination to explicitly share his motivation for malingering, which would of course defeat the purpose.

Methods for the assessment of malingering in general should be useful in detecting feigned homicidality [1]: namely, inconsistencies (1) in the patient’s self-reports of homicidal thoughts (threats); (2) between what the patient reports and what others observe and report; (3) in observations concerning the “symptoms” themselves, in this case the patient’s potential for homicide; (4) between the patients’ self-report and performance on psychological tests (or mental status exam); and (5) in the patient’s report of homicidal threats or thoughts and how actual threats with serious intent are manifested. The task is challenged by the fact that the patient only needs to express homicidal ideations to arouse concern; he need not necessarily malinger a complex condition such as psychosis.

The guidelines proposed by Borum and Reddy [2] are useful in the assessment of risk of homicidality. As in the case of Mr. B, they proved useful in highlighting the discrepancies in his history and the assessment of the overall risk. Moreover, due to safety concerns, it is often difficult to entertain the possibility of feigning symptoms when the risk of homicidality is assessed to be high. However, when the risk is determined to be low, the third dimension, i.e., feigning of homicidality can be further explored, as highlighted in the case of Mr. B. Although it is not always possible to gain knowledge of the potential incentives, an active search may prove to be useful. The areas that may assist in the determination of malingering include (1) a thorough history from the patient—it may even be helpful to periodically reassess the patient and be cognizant of any discrepancies in the narration over time; (2) thorough collateral information—this can prove to be very helpful, especially when obtained from multiple sources; refusal to provide any collateral source and lack of cooperation during the diagnostic or treatment process may also point towards an ulterior motive; (3) an active inquiry regarding the pertinent motives—financial constraints, lack of housing, and legal issues are especially prevalent in certain areas; and (4) legal inquiry—certain states provide an easy to access legal history of their clients, which may disclose any legal matter that the patient might try to avoid or delay by staying in the hospital.

### 3.5. A Multi-Disciplinary Approach

Cases with possible legal implications should involve a multidisciplinary approach to minimize risk and improve quality of assessment and management. Obtaining health records from other medical facilities, reviewing prior medical records, and obtaining collateral information from family members can yield vital sources of information in such cases. Ruling out possible medical issues or lies, such as was done in the Case 1 example above, can provide useful pieces of information in support of malingering. Medical providers may need to involve the ethics committee of the hospital, or consult with the legal department if they are unsure about addressing the legal aspects in these cases, such as in the context of duty to warn, and possible implications of discharging such a patient from the emergency room.

### 3.6. Duty to Protect

In the landmark ruling of Tarasoff v. The Regents of University of California [7], the court ruled that therapists need to take reasonable actions to protect potential victims of possible harm from their dangerous patients. When a patient makes a verbal threat against an identifiable victim, and the provider assesses such threat to be credible, the provider is directed to warn potential victims, inform the authorities, and/or take other necessary action to protect the life of potential victims [10]. Different states in the U.S. have adopted different standards in this area. While some states recommend a duty to warn potential victims, other states recommend a duty to take all necessary steps in addition to warning potential victims, to save lives. Some U.S. states mandate while others merely permit a breach of patient–provider confidentiality to carry out the established duty to warn/protect. While duty to warn is an accepted exception to confidentiality in most countries, only recently has it gained a legal precedent or background in other developed countries. [11]

## 4. Conclusions

We summarize the recommended steps for the evaluation and management of feigned homicidality in Table 2. 

With the use of two case examples, we highlighted the assessment and management of feigned homicidality with an emphasis on the risk of verbal threat, assessment of the seriousness of the risk, differentiation from pseudologia fantastica, understanding duty to protect, and using a multidisciplinary approach.

We propose the addition of the assessment of malingering to the risk assessment for homicidality, especially in cases where there are enough reasons to suspect it. For example, a thorough screening for inconsistencies in the narration and presentation as well as potential external incentives for feigning homicidal symptoms may be included while using Borum and Reddy factors (expanding the acronym to ACTION). Other scales may be developed to include this dimension of homicidality.

## Figures and Tables

**Table 1:** Summary of pertinent findings in the two cases that point towards a possibility of feigned homicidal ideations.

CASE 1	CASE 2
History of substance abuse and legal problems	History of substance abuse
Vague symptoms	Symptoms changed based on circumstances
Uncooperative with detailed examination	Lack of clear plan, means, or history of violence
Story changing over time	Inconsistencies in history
Medical examination did not match history	Irritable when confronted with inconsistencies
Collateral information from family contradicted patient’s story	Collateral information from significant other confirmed that they were feigning psychiatric symptoms
Clear motive to stay inside the hospital and avoid legal consequences during the time of stay	Clear motive to feign symptoms to avoid homelessness and gain a place to stay

**Table 2:** Steps recommended towards the evaluation and management of feigned homicidality.

Inquiries for the evaluation of the verbal threat	Is the patient dangerous to others? Is the danger due to serious mental illness? Is the danger imminent? Are potential victims reasonably identifiable?
Assessing the seriousness of the risk of homicide (ACTION)	Attitude that supports violence Capacity to carry out the threat Threshold crossed towards violence Intent is serious Others in life support violent responses Non-compliant with risk reduction methods
Differentiation from pseudologia fantastica	Pseudologia fantastica manifests as excessive and impulsive lying that is fanciful, done to seek attention or sympathy, and out of proportion to the motive
Assessment of malingering (feigning) when suspected	Locate inconsistencies in the narrative using thorough history Thorough collateral information Active inquiry regarding pertinent motives Legal inquiry
Multidisciplinary approach	Consider consultation with ethics committees, legal counsel, and other specialists as warranted.
Duty to Protect	Take reasonable steps as recommended by the guidelines in that specific jurisdiction

## Data Availability

Not applicable.
